# Virilisation during Pregnancy in a Patient with Metastatic Colorectal Cancer

**DOI:** 10.1155/2012/704039

**Published:** 2012-10-14

**Authors:** F. Conway, S. Jarvis, M. Thornton

**Affiliations:** ^1^General Surgical Department, Wishaw General Hospital, 50 Netherton Street, Wishaw ML2 0DP, UK; ^2^Clinical Laboratories, Wishaw General Hospital, 50 Netherton Street, Wishaw ML2 0DP, UK

## Abstract

This paper describes the case of a 25-year-old woman with virilisation occurring during pregnancy in the presence of metastatic colorectal cancer. Virilisation during pregnancy is rare. The potential causes include adrenal, foetal, or ovarian pathologies. The most common causes during pregnancy are pregnancy luteoma and hyperreactio luteinalis. The incidence of cancer during pregnancy is rare and the incidence of colorectal cancer (CRC) in pregnancy is even rarer. The presenting signs and symptoms of CRC can be confused with symptoms commonly encountered during pregnancy, thereby delaying diagnosis and commencement of treatment. Diagnosis and staging also proves more problematic in the pregnant patient as the usual modalities of colonoscopy with biopsy and imaging with CT are relatively contraindicated. Treatment is dependent on gestational age of the foetus. There is currently no agreed best practice as to the role of prophylactic oophorectomy in the prevention of metachronous ovarian metastases. Surgical and adjuvant treatments have implications for females of child-bearing age.

## 1. Introduction

This paper describes the case of a 25-year-old woman with virilisation occurring during pregnancy in the presence of metastatic colorectal cancer.

The incidence of cancer during pregnancy is rare and ranges from 0.07% to 0.1%. The incidence of colorectal cancer (CRC) in pregnancy is reported at 0.002% [1]. The presenting signs and symptoms of CRC can easily be confused with symptoms commonly encountered during pregnancy, thereby delaying diagnosis and commencement of treatment. Diagnosis and staging also proves more problematic in the pregnant patient as the usual modalities of colonoscopy with biopsy and imaging with CT are relatively contraindicated. Treatment is dependent on gestational age of the foetus.

Reports of ovarian metastasis from CRC primaries in the literature vary between 4 and 30.8% [2]. There are no current national guidelines for the role of prophylactic oophorectomy in the prevention of metachronous ovarian metastases. Surgical and adjuvant treatments have obvious implications for females of child-bearing age. 

Virilisation during pregnancy is rare. The potential causes include adrenal, foetal, or ovarian pathologies. The most common causes during pregnancy are pregnancy luteoma and hyperreactio luteinalis [3].

## 2. Case Report

This 25-year-old Caucasian woman presented with severe abdominal pain and symptoms of intestinal obstruction at 37-week gestation. During her 2nd and 3rd trimesters she developed facial hirsutism, deepening voice, facial acne, and clitoromegaly. Her past medical history included recurrent UTI as a child, asthma for which she had received oral steroids intermittently, and 4 laparoscopies (aged 17–24 years) at which endometriosis and polycystic ovarian syndrome was diagnosed. 

Her biochemistry revealed testosterone 45.0 nmol/L (normal range 1–3.2), SHBG (sex-hormone-binding globulin) 391 nmol/L (range 20–155), androstenedione > 200 nmol/L (range 0.6–80), 17 OH-progesterone 295.0 nmol/L (range < 13.0), CEA 1.8 ng/mL (range < 6.0), and CA125 389.7 U/mL (range < 35.0)

**Figure 1:**
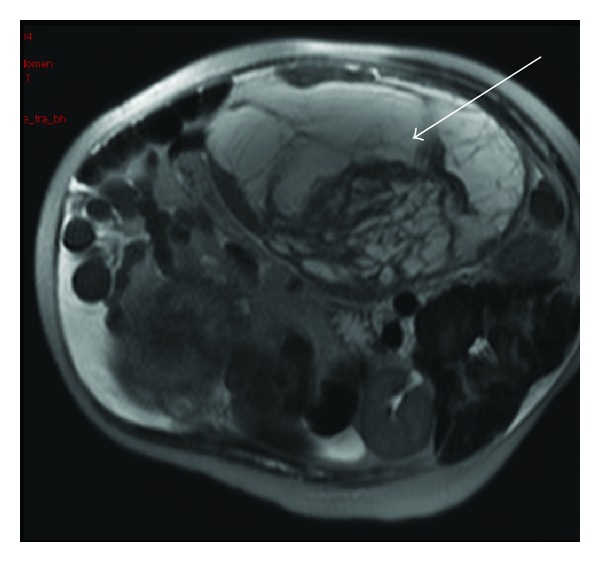
MRI abdomen, arrow indicating complex ovarian mass.

**Figure 2:**
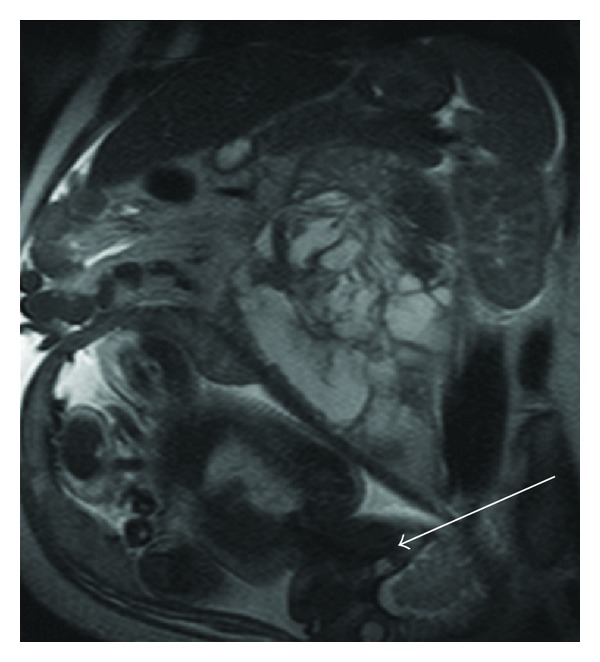
MRI abdomen, arrow pointing to gravid uterus.

She underwent a pelvic ultrasound that revealed a large complex left-sided pelvic/abdominal mass. Subsequent MRI highlighted a large ovarian malignancy (Figures 1 and 2). A Caesarean section was performed along with left salpingo-oophorectomy, appendicectomy, right ovarian, and omental biopsies. She delivered a healthy male who has been karyotyped as XY. 

Histopathology confirmed a left ovarian moderately differentiated adenocarcinoma measuring 265 × 220 × 125 mm, a likely metastatic deposit from a gastrointestinal primary. Following delivery, her androgen profile returned to normal. Staging CT chest, abdomen, and pelvis revealed no new pathology. At colonoscopy a moderately differentiated invasive caecal adenocarcinoma was biopsied. Following a literature search, MDT discussion, and involvement of Scottish oncology and fertility experts she proceeded to a right hemicolectomy, right salpingo-oophorectomy, and peritoneal biopsies. Her disease was staged pT3, N2, M1 (6 of 19 nodes were involved and there was extramural venous invasion). The right ovary, resection margins, and peritoneum were disease-free. The tumour was oestrogen and progesterone receptor negative. She was referred for adjuvant chemotherapy. Her testosterone levels did have a transient rise postoperatively but returned quickly to within normal limits. The affected ovary has been further examined to determine whether it was tumour or ovarian tissue that was responsible for her abnormal biochemical profile.

## 3. Discussion

Colorectal cancer in pregnancy is rare and can result in serious morbidity/mortality for both mother and foetus. 

### 3.1. Aetiology

It has been suggested that this patient population is more at risk with 16% having predisposing conditions including HNPCC, FAP, Peutz-Jeqher's syndrome, and a history of IBD and 23% has a family history of CRC [4]. 

During pregnancy oestrogen, progesterone, and prolactin levels all increase thereby potentially contributing to disease progression. 30–67% of CRC tumours are oestrogen receptor (ER) positive and 10–100% are progesterone receptor (PR) positive [5]. 

Eberhart et al. studied the role of cyclooxygenase-2 enzyme in pregnancy and colorectal cancer and concluded that 50% of colonic adenomas and 80–85% of colonic adenocarcinomas showed increased expression of COX-2 enzyme [6].

### 3.2. Diagnosis and Staging

Common presenting signs and symptoms of CRC include abdominal pain, abdominal mass, altered bowel habit, anaemia, and nausea and vomiting; all of which can be attributed to the pregnancy itself. Rectal bleeding is also common in pregnancy secondary to haemorrhoids.

Endoscopy is preferentially deferred until the second trimester if possible. Patients should be fully informed of risks to the foetus prior to commencing colonoscopy. Maternal oxygen therapy, blood pressure monitoring, and foetal cardiac monitoring are recommended to identify any signs of foetal distress [7]. Complications include placental abruption from mechanical pressure applied to uterus and foetal injury secondary to maternal hypoxia or hypotension during the procedure [8].

Serum CEA is measured as a baseline preoperatively and throughout followup to monitor for disease recurrence in CRC. CEA levels can be normal or marginally elevated during pregnancy [9].

CT scanning is routinely performed in the staging of CRC to identify the extent of local disease and distant metastases. This helps determine surgical and neoadjuvant/ adjuvant therapies best suited to patient disease pattern. CT scans are contraindicated in the pregnant patient due to radiation teratogenicity, in particular in the first trimester [10, 11]. Abdominal ultrasound can be used for detecting macrometastatic liver lesions with a sensitivity of 75% [12]. MRI is a further modality that does not expose the foetus to ionizing radiation.

In a literature review, Bernstein et al. discovered that 85% of CRC in pregnant females were below the peritoneal reflection and 60% of all cases presented with Duke C or more advanced disease [13].

The incidence of ovarian metastases from colorectal primaries is quoted in the literature as 3–8% in nonpregnant patients but may be as high as 25% in pregnant patients [14, 15]. CRC with ovarian metastases is associated with a poor prognosis with median survival following resection 6–18 months [16]. The incidence of metachronous ovarian tumours has been reported at 1.3–6.6% [17, 18] raising the debate as to whether prophylactic bilateral salpingo-oophorectomy is clinically indicated and its impact on overall survival.

### 3.3. Virilisation in Pregnancy

As mentioned previously, the causes of virilisation during pregnancy can be ovarian, foetal, or adrenal in nature. Ovarian causes include primary malignancy which is rare, polycystic ovarian syndrome (PCOS), luteoma, and hyperreactio luteinalis (HL). Androgen production is usually in proportion to ovarian size in luteomas and HL [19]. There are certain protective mechanisms in place for both mother and foetus against virilisation. In mothers this includes high levels of SHBG, placental aromatase, and high progesterone levels. The foetus is protected in part by placental aromatase that turns androgens into oestrogens and in part by SHBG [20]. Despite these protective mechanisms, if the level of androgenisation is high enough, the female foetus can be affected.

### 3.4. Treatment

The aim is to start treatment for the mother as early as possible and to deliver the baby as early as possible. The timing of surgery is usually determined by stage of pregnancy. In the first 20 weeks of pregnancy, the recommendation is for termination in favour of surgical resection to prevent significant disease progression during the latter half of pregnancy. In pregnancies beyond 20-week gestation, surgery is delayed until foetal lung maturation has occurred [21]. At this stage, surgical resection can be carried out at Caesarean section or delayed to await uterine involution and resolution of vascular engorgement of pregnancy [22]. All delays in surgical resection should be extensively discussed with the patient identifying risks of disease progression in the interim.

Advanced rectal tumours are frequently treated with radiotherapy prior to resection but pelvic radiotherapy is not recommended in the pregnant patient due to potential risks to the foetus. Likewise neoadjuvant chemotherapy should be considered carefully prior to administration in the pregnant patient with maximal complications being reported in 3–12-week gestation [23]. Chemotherapy in the 2nd and 3rd trimester has been reported as safer but there have been recognised complications such as an increase in the incidence of intrauterine growth retardation and prematurity [24]. 

Ovarian metastasis is a poor prognostic factor with average survival of 17.8 months (range 1 to 86 months) [25]. Furthermore, in cases of colorectal cancer with ovarian metastasis, there does not appear to be any agreed best practice as to whether bilateral oophorectomies should be performed in order to improve overall survival rates. Dae et al. recommend performing bilateral oophorectomies as the incidence of bilateral involvement is significant [26]. It also eliminates the risk of developing primary ovarian cancer in the future. Lee et al. carried out a retrospective study between 1996 and 2003 that concluded oophorectomy prolonged survival in patients with ovarian metastases by almost 10 months [27]. Omranipour et al. did not support the treatment of prophylactic bilateral oophorectomy as they did not find the incidence of synchronous and metachronous ovarian tumours to be high and concluded that overall long-term survival was unaffected [28, 29]. It would appear that this is an area for future research.
